# Environmental drivers of Odonate assemblages across biogeographic provinces in the Mexican Transition Zone

**DOI:** 10.3897/BDJ.14.e189309

**Published:** 2026-06-16

**Authors:** Josué Dolores Silva-Hurtado, Ana Paola Martínez-Falcón, Irene Goyenechea, Sergio López-Mendoza, Pablo Octavio-Aguilar

**Affiliations:** 1 Universidad Autónoma del Еstado de Hidalgo, Mineral de la Reforma, Mexico Universidad Autónoma del Еstado de Hidalgo Mineral de la Reforma Mexico; 2 Universidad de Ciencias y Artes de Chiapas, Chiapas, Mexico Universidad de Ciencias y Artes de Chiapas Chiapas Mexico

**Keywords:** conservation, damselflies, dragonflies, diversity, environmental variation, species richness

## Abstract

This study shows how the different environments and biogeographical provinces in the Mexican Transition Zone influence the structure of odonate communities. Through sampling conducted in 2024 across lentic and lotic habitats in a representative area where four biogeographic provinces converge, habitats were compared by recording physicochemical water parameters and climate conditions. The results revealed that species richness did not vary significantly between habitat types (lentic vs. lotic), but showed marked differences at the biogeographical scale, with the arid and semi-arid zones being the most diverse and high elevations having the lowest species richness. Beta diversity analysis indicated higher turnover than nestedness, suggesting that each region hosts a unique fauna adapted to specific conditions. Environmental variables, such as air and water temperatures and humidity played a crucial role, differentially affecting suborders: Anisoptera showed a greater capacity for dispersal and were more affected by landscape-level, while Zygoptera exhibited higher sensitivity to local factors. We concluded that environmental heterogeneity and biogeographical factors are determinants of odonate biodiversity in the region, with Anisoptera following a classical hierarchical community assembly mechanism and Zygoptera is being influenced and limited by the filter created by environmental factors. It is imperative to implement conservation strategies that protect connectivity and integrity of waterbodies to preserve this unique biological heritage.

## Introduction

Freshwater habitats present two main different water systems, lotic and lentic waters, which differ in their environmental and spatiotemporal settings (Seidu et al. 2019). They are differentiated by physicochemical parameters of the water such as turbidity, organic matter, pH, dissolved oxygen and flow regimes. These water systems support heterogeneous environments that provide favourable conditions for communities, including the Odonata (Scheffer et al. 2006).

Lentic environments tend to be geologically less predictable over time and this phenomenon has tended to exert pressure on species to adapt faster in order to be able to disperse and persist. Ponds are important refuge for Odonata conservation because they are relatively isolated and sometimes show greater heterogeneity in species assemblages. Variability in pond isolation tends to attract good disperser Odonata such as species from the Libellulidae family (Corbet and May 2008). In contrast, lotic systems are characterised by dense vegetation cover, supporting greater abundance and species richness of zygopterans than to anisopterans, as a result of their plant association containing dense vegetation cover along the fringes of the systems. The suite of varying microhabitat complexity along lotic systems has contributed to species heterogeneity, largely dominated by the Zygoptera suborder (Maltchik et al. 2010).

Community assembly is influenced by different processes at a wide range of spatiotemporal scales. Identifying the factors that determine local and regional species assemblages remains a central challenge in ecology. Abiotic factors (humidity, temperature and others) are often thought to shape the limits of species distributions. Belyea and Lancaster (1999) referred to this as “environmental filtering” hypotheses, whereas community assembly at local scales is often thought to be driven by biotic factors (mutualism, niche competition, predation and resource consumption), in which biotic and abiotic factors act as “filters” to exclude potential species in the community. Depending on their strength, the benefits of non-filtering interactions increase species’ fitness and their ability to survive and be part of ecological networks (Baecher 2024). The most common theory explains that this assembly of species passes through an environmental (abiotic) filter and a biotic filter (HilleRisLambers et al. 2012).

All species belong to a regional pool constrained by historical processes and, during filtering, a subset of the regional pool (defined by dispersal abilities) is available for colonisation of a local site.

According to the neutral theory proposed by Hubbell (2001), spatial limitation of species (movement) is a key factor for maintaining biodiversity, rather than local environmental adaptation. Local and regional diversity may be determined by the dispersal and ecophysiological ability of individuals to occupy and co-exist in different spatial and temporal scales (Hanski and Gilpin 1991, Urban 2004). According to Heiser and Schmitt (2009), there are differences in dispersal ability and these are related to dependence on temperature, climate and the environment. Odonate adults, due to their aerial-terrestrial habits, exhibit fewer restrictions to variations in physicochemical water parameters, having a greater association with solar radiation, temperature and air humidity (D'Amico et al. 2004, Monteiro-Júnior et al. 2014, Oliveira-Junior and Juen 2019).

Odonates are a good example of environmental filtering and changes in assemblages, due to their important role in ecosystems, both as generalist predators and as prey and as indicators of the environment and water quality (González-Soriano et al. 2011) and wide distribution throughout the world. Although they have an affinity for areas with high humidity and temperature, they have managed to adapt to many other habitats, such as phytotelmata and sulphurous and brackish waters, primarily in lentic and lotic environments (González-Soriano 1997, Needham et al. 2000). This has led to the development of survival or colonisation strategies and the diversification of odonates into specialist and generalist habitat species, endemic species and widely distributed species amongst others (Balvanera et al. 2006, Abbott 2011, Cardinale et al. 2012). In Mexico, this diversity of odonates is reflected in the 374 species present in the country (Paulson et al. 2025) out of the 6,468 species registered worldwide (Paulson et al. 2025).

Physical structuring, air and limnological variables, particularly humidity and temperature, are important factors influencing the diversity and distribution of odonates; however, they are also influenced by other larger scale abiotic factors, such as biogeographic history and, on local scales, by factors such as the environmental variables of water (pH, conductivity, dissolved solids, pollution, disturbance and presence of vegetation amongst many others). Multiple studies conducted worldwide show how various environmental factors affect dragonflies and damselflies (Juen et al. 2006,Kadoya et al. 2007,Monteiro-Júnior et al. 2014,Brasil et al. 2017). Some examples are the study by Oliveira-Junior and Juen (2019), who observed that, in the case of anisopterans, environmental factors affect the variation in the community. However, this group has greater dispersal capacity, allowing it to avoid adverse conditions. In contrast, zygopterans have lower dispersal capacity, depending more on local conditions (physical and chemical parameters of the water, flow structure and vegetation cover), making them habitat specialists.

Changes or modifications due to anthropogenic activities modify the taxonomic composition of odonates, since adult odonates have very specific habitat requirements with respect to the physicochemical quality of the water and riparian vegetation, with water quality being a key factor. It has been observed that species richness increases with higher dissolved oxygen, water temperature and vegetation cover, while it decreases with increasing water level, pH and total dissolved solids (Ishak et al. 2021). Gómez-Anaya et al. (2023) found that areas with land-use modifications and sewage discharges with high concentrations of nitrites and ammonium modify factors such as pH, oxygen and conductivity, to the detriment of odonate diversity. Renner et al. (2022) found that anthropogenic modifications are shifting species assemblages by favouring common species at the expense of rare ones, suggesting that the increase in common species reduces available niche space for weaker competitors, while the richness of rare species declines and these are strictly associated with native grasslands and negatively impacted by agriculture and salinity. Ultimately, human-driven environmental changes have increased the prevalence of common species, disrupting the biome’s original balance and threatening the survival of regionally rare specialists.

Mexico has an interesting and significant biogeographic history, as the country contains a high level of biodiversity that reflects its varied vegetation, climate and biological groups. The country is part of the Mexican Transition Zone (MTZ) where the great exchange of species between North America and the south takes place (Morrone 2019). The State of Hidalgo is located in the central part of Mexico and is part of the MTZ. The biogeographic component is particularly notable, being composed of four biogeographic provinces: the Veracruzan Province (VE), the Sierra Madre Oriental (SMO), the Trans-Mexican Volcanic Belt (TMVB) and the Chihuahuan Desert (CHD) which, together with the environmental heterogeneity, large freshwater reserves and multiple waterbodies, contribute to a significant odonate diversity in the State (Escoto-Moreno 2015, Morrone et al. 2017).

Hidalgo is one of the States with the greatest species richness. Various studies have been carried out in different regions of the State, contributing records from areas in the northern Sierra of Hidalgo and the Mezquital Valley (Novelo-Gutiérrez and Peña-Olmedo 1991, Peña-Olmedo and Novelo-Gutiérrez 1993, Peña-Olmedo 2001) and from the north-western region of the State (Gómez-Anaya et al. 2000, Novelo-Gutiérrez et al. 2002). Other species records have been provided by Behrstock 2005 and Behrstock et al. 2007. Some odonate studies have taken a biogeographic approach, setting out to analyse the affinities that odonates maintain with various biogeographic histories in the State (Novelo-Gutiérrez and Peña-Olmedo 1991, Alonso-EguíaLis et al. 2002, Escoto-Moreno 2015). Another important research of Odonata diversity in Hidalgo State was made by Novelo-Gutiérrez et al. (2002) who used odonate larvae as indicators of environmental quality, this study pioneering the analysis of odonate community structure in specific regions, highlighting the previous lack of measurements such as the number of species (species richness), proportional abundances and their correlation with environmental variables (temperature, pH, dissolved solids, and salinity, amongst others).

The objective of this study is to analyse differences in species composition between lentic and lotic waterbodies and their relationship to available habitats in the biogeographic provinces of the State of Hidalgo. The research questions are: i) Are there differences in the odonate species assemblage, measured by alpha and beta diversity, between the biogeographic provinces of the State of Hidalgo; ii) between lentic and lotic water bodies;and iii) How do abiotic parameters influence the distribution of odonates in lentic and lotic habitats within the biogeographic provinces?

We hypothesise that environmental filtering, rather than historical barriers or dispersal limitation, drives Odonata community structure in the study area, which lies within a small State where four biogeographical regions converge. We predict that community assemblages will vary significantly amongst the converging biogeographic provinces in response to their distinct abiotic profiles. Richness and diversity are expected to peak in the high temperature and high humidity environments of the SMO and VE, while reaching their lowest levels in the arid zone from CHD and high altitudes of TMVB. Consistent with this environmental and spatial gradient, the most distant provinces (CHD and VE) will display markedly distinct species compositions.

We expect that the main determinants of interprovincial differences will be some species of the suborder Anisoptera, such as those in the families Aeshnidae and Libellulidae, because they are found across biogeographic provinces due to their great diversity and broad dispersal capabilities. While Damselflies (Zygoptera) are expected to share species of the family Coenagrionidae across provinces, while other species will be restricted by their limited dispersal capacity. Finally, according with neutral theory, we expect that spatial limitation of species (movement) play a role on regional biodiversity, meanwhile environmental parameters may determinate local diversity patterns.

## Material and methods

### Study area and fieldwork

**Figure 1.**
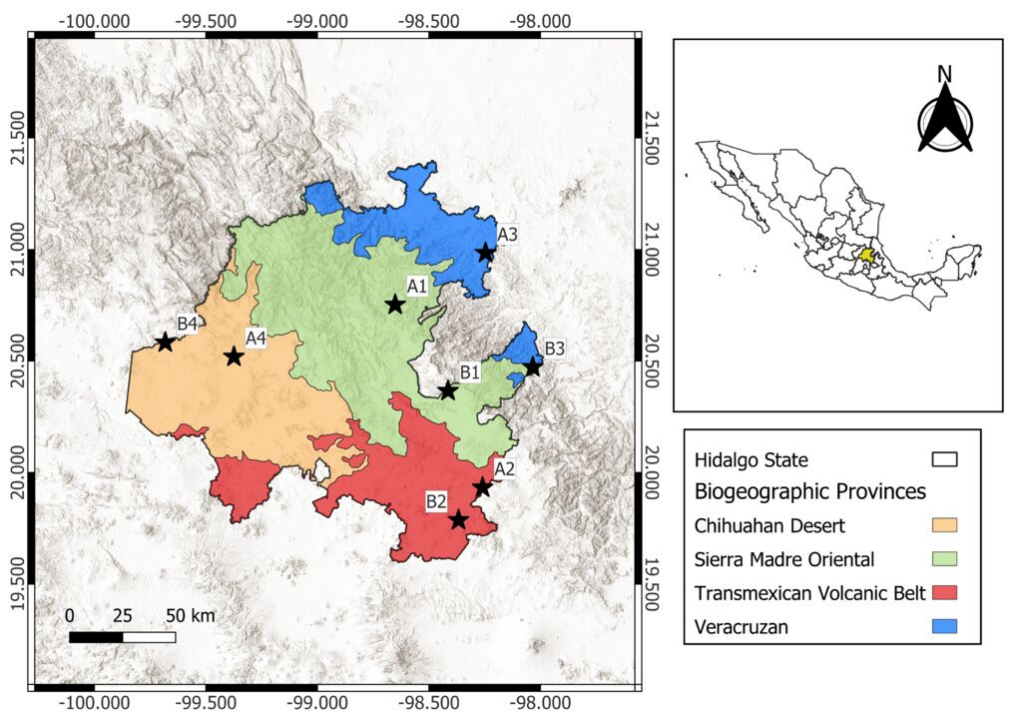
Map of the State of Hidalgo, Mexico showing biogeographic provinces and sampling sites (black stars).

The State of Hidalgo is characterised by the convergence of four biogeographic provinces (Morrone et al. 2017 and Morrone 2019), resulting in a diverse array of environmental factors including vegetation, elevation, temperature and precipitation. The description of the sampling locations was based on the characteristics described by INEGI 1995, [Bibr B13917688][Bibr B13917872], Secretaría de Planeación 2011 (Table 1, Fig. 1).

For the collection of odonates, two sampling localities were selected from each of the four biogeographic provinces (eight in total). Within each locality, four lentic habitats (pools, temporary ponds and drainage ditches) and four lotic habitats (rivers, streams and creeks) were selected, resulting in 16 sampling points for each biogeographic province per season, for a total of 64 sampling points. Visits to each location were conducted in March (dry season) and September (rainy season) in 2024 to capture as many species as possible at the respective sites. This also enabled changes in habitat and species composition to be observed. March is the driest month, with the least rainfall, while September has the highest rainfall and abundance of adult dragonflies, since many species reproduce at this time (Paulson and Haber 2021) and a greater probability of spotting migratory species (Paulson 2009).

Collecting took place during the odonates activity period, from 10 a.m. to 2 p.m. (four hours), yielding a total of 64 hours of sampling effort for each sampling site, which was carried out by one person. The collection of odonate individuals was authorised by a Scientific Collection License by Research Area for Researchers and Scientific Collectors Linked to Research Institutions, with official number SPARN/DGVS/03335/24.

The collected adult specimens were placed in a freezer to reduce their temperature and euthanise them, immersing them in 100% acetone for at least 24 hours to maintain their colouration (Morón and Terrón 1998).

Specimens were identified with the help of specialised literature (Garrison 1990, [Bibr B13916383][Bibr B13916392], [Bibr B13916483][Bibr B13916400][Bibr B13916667], Garrison and Ellenrieder 2022). Once they had been identified, a taxonomic list of the collected species was compiled, based on the classification criteria proposed by Paulson et al. (2025). The specimens were then deposited in the Ecology of Communities Laboratory at the UAEH Biological Research Center.

### Environmental data collection

To address the habitat preferences of the odonates, environmental data were collected from each of the sampling locations using the Extech (445702) 3ZH92 hygrothermometer to measure the maximum (TMAX) and minimum (TMIN) ambient temperature and the maximum (HMAX) and minimum (HMIN) percent ambient humidity. A HI98130 pocket meter with automatic calibration was used to compensate for temperature when measuring pH and total dissolved solids (TDS). This tool was used to measure water temperature (TA), pH, TDS particles per million (ppm), conductivity (COND) and percent salinity (SAL). Each waterbody was classified as a lentic environment (pools, temporary puddles, ponds and drains) or a lotic environment (flowing rivers, streams and creeks) (Novelo-Gutiérrez et al. 2002 andGómez-Anaya et al. 2023) (Table 3).

### Data analysis

#### Alpha diversity

Sample coverage was calculated to determine the completeness of the inventory by biogeographic province and habitat. Alpha diversity was estimated amongst biogeographic provinces, habitat types and for each suborder of the Odonata community, using order ^0^*D*, which measures species richness and ^1^D, the Shannon index, which also accounts for the abundances of the species. These parameters were compared amongst biogeographic provinces and habitats using 95% confidence intervals calculated with the online programme iNEXT (Chao et al. 2016). Additionally rank-abundance curves were constructed to assess the numerical importance of species in each of the biogeographic provinces.

#### Beta diversity

To compare beta diversity amongst biogeographic provinces and habitats, we used the script of Carvalho et al. (2011) which was calculated for each suborder using the R 4.4.0 programme. According to the method of Baselga (Baselga 2009), total dissimilarity (βcc) is one minus the Jaccard coefficient of similarity. This total dissimilarity can be divided into two components; dissimilarity due to turnover (β.3; species replacement between communities) and differences in richness or nestedness (βrich; gain or loss of species between communities). The turnover component may be due to environmental filtering or spatial and historical factors and constraints. Neverthless, it will be independent of differences in the number of species per site. In contrast, nestedness is taken as richness gradients where the dissimilarity between communities originates due to a different number of species, related to extinction colonisation dynamics (Moreno 2001, Calderón-Patrón and Moreno 2019).

#### Environmental relationships

Databases containing environmental and species information were created. A PERMANOVA, based on Jaccard distance, was conducted using the Primer v.7 programme (Clarke and Gorley 2015), to find whether there are significant differences between biogeographic provinces and habitats (Anderson 2001). The results were graphed using non-metric multidimensional scaling (NMDS) with the help of the Past 4.17 programme (Hammer et al. 2001).

Subsequently, a paired test analysis was carried out to compare biogeographic provinces, using a Bonferroni correction to adjust the significance level to a value of p < 0.05 and the variation of each biogeographic province was assessed by a multivariate permutation analysis of dispersal (PERMDISP = P_perm_) (Anderson 2004), based on the distance of the Jaccard index from the group mean.

Finally, to address directly the possible influence of the environmental variables and reduce the dimensionality of these, a principal component analysis (PCA) (Hotelling 1933) was used with a variance-covariance matrix and the error was extracted using a normalised varimax rotation, which guarantees the orthogonality and multinormality of the environmental data.

With the environmental matrix cleaned by factor analysis, a canonical correspondence analysis (Ter Braak 1986) was performed to establish linear relationships amongst the presence of species by group, the environmental variables and the cases per environment.

These analyses were carried out by suborder and for the entire dataset.

## Results

### Taxonomic diversity

A total of 380 individuals distributed in six families, 22 genera and 51 species were collected (Table 2) Twenty-two species, three families and 15 genera were identified for the suborder Anisoptera, with Libellulidae being the most represented family (16 species). The sample from suborder Zygoptera comprised three families, seven genera and 29 species, with the family Coenagrionidae being the most represented (21 species). The province with the highest Odonata species representation was the CHD with 27 species, followed by the VE and the SMO with 22 and 20 species, respectively; the province with the fewest species was the TMVB with 10 (Table 2).

Most notable are the records of species with restricted distribution, such as *H. infecta*, *A. barretti*, *A. percellulata* and *C. vibex* and migratory species such as *A. junius*, *E. umbrata*, *P. flavescens*, *S. corruptum* and *P. longipennis* (Burmeister, 1839), the latter being a new record for the State of Hidalgo.

With this new record of *P. longipennis*, the State of Hidalgo has 151 species, approximately 42% of the country’s odonate species (Paulson et al. 2024, Paulson et al. 2025). This makes Hidalgo the State with the fifth highest Odonata diversity in Mexico.

### Alpha and beta diversity

Based on the sample coverage, the inventory completeness by biogeographic province was 0.99 for the SMO, 0.95 for the TMVB, 0.95 for the VE and 0.93 for the CHD.

**Figure 2.**
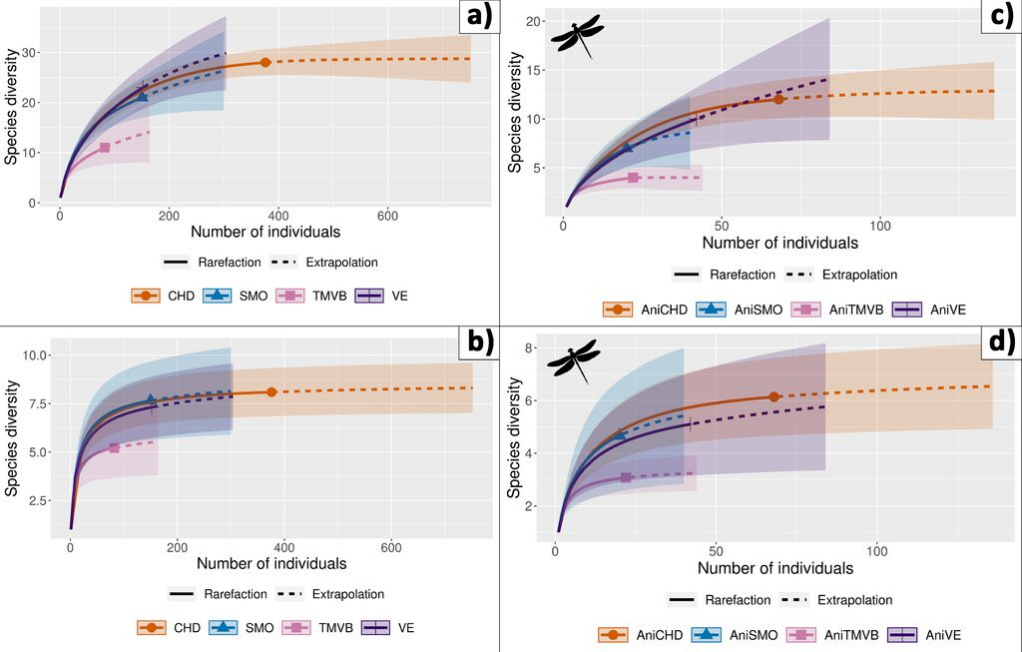
Species richness curves of order ^0^D and ^1^D by: **a, b**) biogeographic province; **c, d** Anisoptera and biogeographic provinces.

The CHD Province presented the highest ^0^*D* species richness; however, no significant differences were observed between the SMO (^0^*D* = 20, I.C = 15.78;^1^*D* = 14.62, I.C = 4.48; ^2^*D* = 11.84, I.C = 3.11), the VE (^0^*D* = 22, I.C = 13.47;^1^*D* = 13.35, I.C = 3.74; ^2^*D* = 9.17, I.C = 2.82) and the CHD (^0^*D* = 27, I.C = 5.3; ^1^*D* = 16.37, I.C = 2.2; ^2^*D* = 11.72, I.C = 1.85), as their 95% confidence intervals overlapped. The TMVB (^0^*D* = 10, I.C = 7.59; ^1^*D* = 6.77, I.C = 2.57; ^2^*D* = 5.34, I.C = 2.08) is significantly lower in species richness than the CHD (Fig. 2). Contrary to what we believed, there were no significant differences in species richness between lentic (^0^*D* = 40, I.C = 6.93; ^1^*D* = 10.13, I.C = 2) and lotic (^0^*D* = 34, I.C = 11.5; ^1^*D* = 8.6, I.C = 1.52) habitat types.

Significant differences were found for the suborder Anisoptera in ^0^*D* between the CHD compared to the VE and TMVB (Fig. 2). In ^1^*D*, the significant differences were between the CHD and TMVB, while no differences were found for the suborder Zygoptera between the species richness (^0^*D*) of the provinces and the abundances (^1^*D*). For ^2^*D*, significant differences were found between the CHD and TMVB. The curves showed differences (Fig. 2).

**Figure 3.**
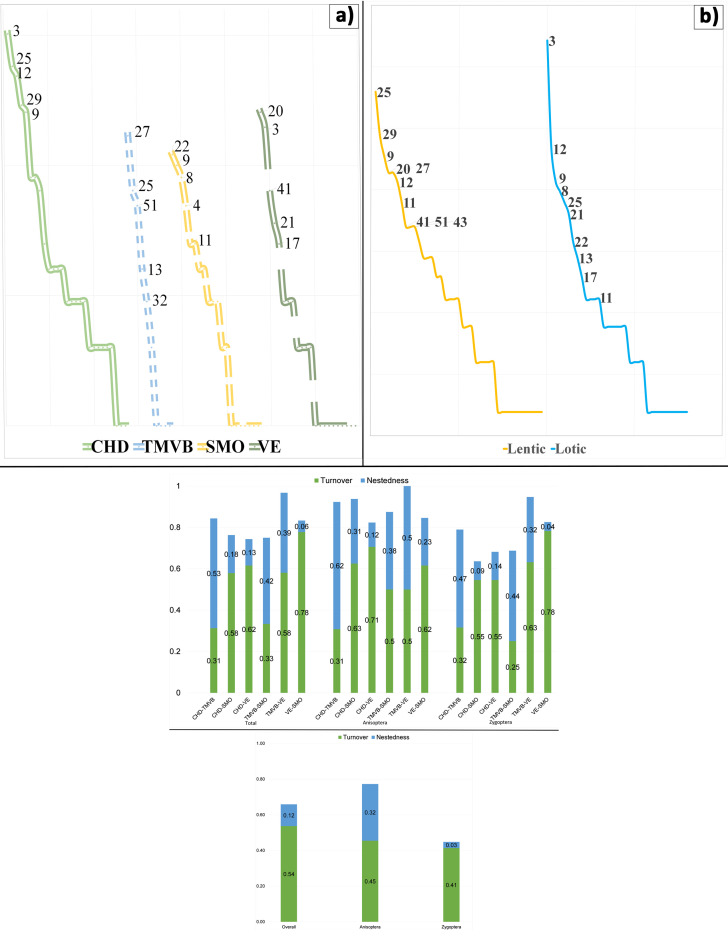
Rank-abundance curves by a) biogeographic province; b) habitat. The number on each curve corresponds to the five and ten most abundant species, keys to Odonata species are in Table 2. Beta diversity amongst biogeographic provinces and habitat type.

The rank-abundance curves revealed that the species dominating the biogeographic provinces belong to the suborder Zygoptera and families Coenagrionidae and Calopterygidae. For the suborder Anisoptera, species collected belong to the family Libellulidae. The dominant species in each province are *H. calverti* in the CHD, *I. demorsa* in the TMVB, *A. ulmeca* in the SMO and *A. sedula* in the VE (Fig. 3). The dominant species across provinces reflect turnover in species composition and abundances amongst provinces. By habitat type, *E. praevarum* dominated the lentic systems and *H. calverti* the lotic systems (Fig. 3).

The dissimilarity analysis between biogeographic provinces showed an average value of 0.7. Turnover accounted for a greater proportion of beta diversity, with an average of 0.5, while nestedness had an average of 0.2. In comparisons between provinces, nesting had a greater influence for TMVB–CHD and TMVB–SMO (Fig. 3). The greatest dissimilarities were observed between the TMVB and the other provinces. This may be due to the TMVB presenting high differences in species composition. The highest dissimilarity value was observed in the TMVB–VE pair (0.84), while the lowest dissimilarity was found in the CHD–VE pair (0.74) (Fig. 3).

For the suborder Anisoptera, total dissimilarity was 0.91, turnover was 0.54 and nestedness was 0.36. The suborder Zygoptera had a lower total dissimilarity of 0.76, with 0.51 turnover and 0.25 nestedness (Fig. 3) .

The number of species shared between the two habitat types was 21, while 18 species were found only in lotic environments and 12 only in lentic environments. The total dissimilarity between lotic and lentic habitats was 0.66; for the suborder Anisoptera, dissimilarity was higher with 0.77; while, for the suborder Zygoptera, the value was lower at 0.45. Turnover predominated in most cases (Fig. 3).

### Environmental relationships

**Figure 4.**
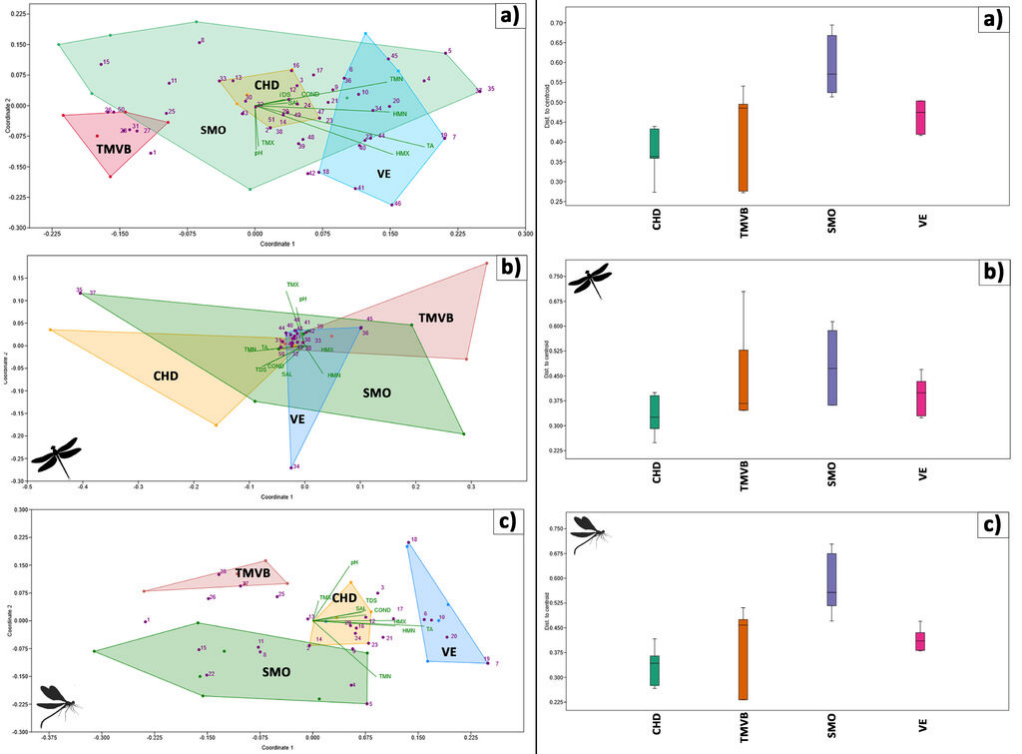
Non-metric multidimensional scaling (NMDS) using the Jaccard index, taking biogeographic province and environmental variables as factors: (TMN = Minimal ambient temperature, TMX = Maximum ambient temperature, HMN = Minimum humidity, HMX = Maximum humidity, TA = Water temperature, TDS = Total dissolved solids, SAL = Salinity, COND = Conductivity) (keys to odonate species are in Table 2); Distance from the centroid for each biogeographic province. a) total, b) Anisoptera, c) Zygoptera.

The results of the NMDS analysis reveal an ordination differential amongst provinces. Mostly, species in the TMVB, CHD and VE remain grouped by the biogeographic province to which they belong, but the SMO is dispersed throughout the graph, overlapping with the other provinces. Some of the environmental variables (TMAX, HMAX, TA) in the VE and parts of the SMO showed a positive correlation, but, for the TMVB and some areas of the SMO, a negative correlation is observed between species and environmental variables (Fig. 4).

Amongst environmental factors, HMX and TA were strongly correlated with Anisoptera species (Fig. 4). For the suborder Zygoptera, species respond positively to environmental variables pH, TMN, TA, HMN and COND (Fig. 4).

A PERMANOVA analysis was carried out using the environmental variables. The biogeographic provinces exhibited significant differences (F = 3.308, p < 0.0001), whereas the habitat types did not (F = 1.307, p = 0.1271). The paired tests between levels also show significant differences in species composition across biogeographic provinces (Table 4). Dispersal in relation to the centroid of each biogeographic province varied significantly (F = 13.99, p = 0.0001) for the general data and for suborder Zygoptera (F = 15.69, p = 0.0001), which had the same pattern and varied significantly (Fig. 4). Suborder Anisoptera (F = 4.867, p = 0.08) did not show significant differences between biogeographic provinces (Fig. 4).

The results of the present study show the strong influence of biogeographic provinces on odonate species assemblages, reflecting each province’s unique environmental characteristics (Fig. 6).

**Figure 5.**
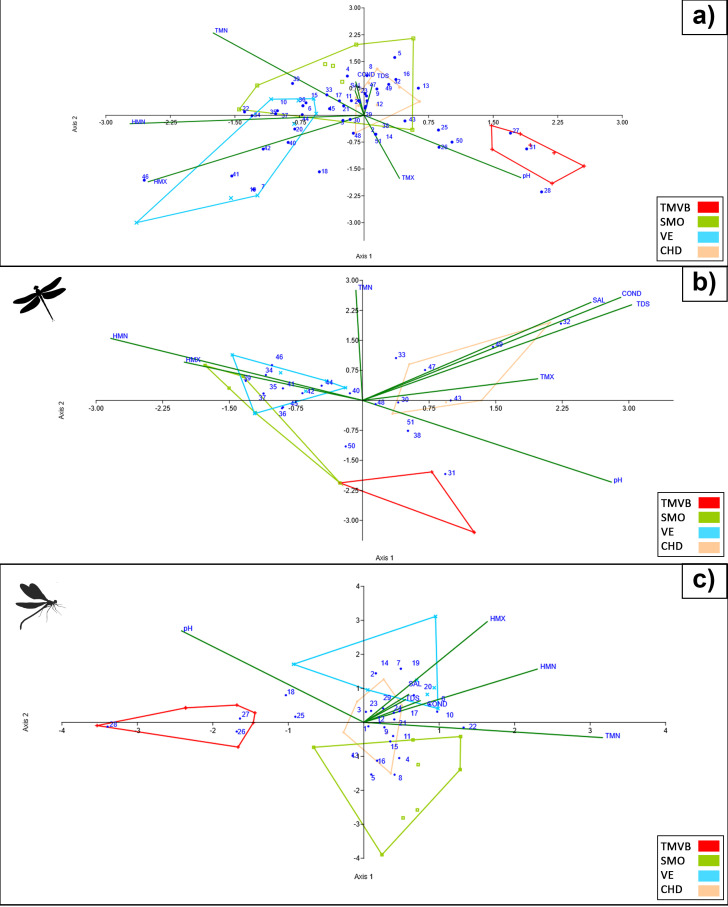
Ordination diagram of the first two axes of the canonical correspondence analysis (CCA) using biogeographic province and environmental variables as factors: a) Total, b) Anisoptera, c) Zygoptera (TMN = Minimal ambient temperature, TMX = Maximum ambient temperature, HMN = Minimum humidity, HMX = Maximum humidity, TA = Water temperature, TDS = Total dissolved solids, SAL = Salinity, COND = Conductivity) (keys to odonate species are on Table 2).

**Figure 6.**
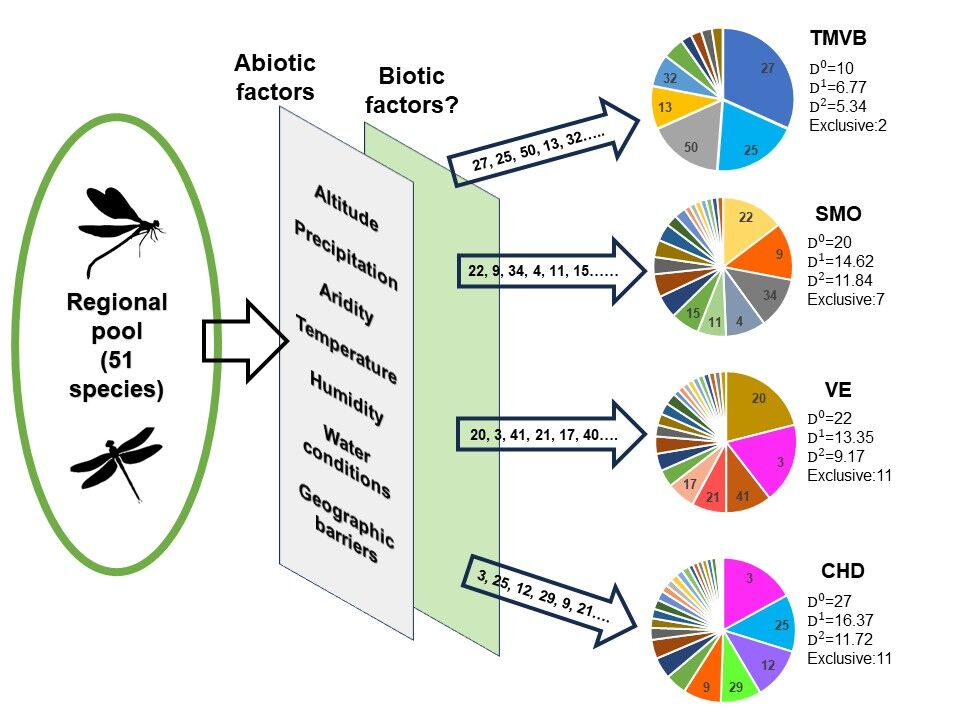
Community assembly of Odonata in the biogeographic provinces in Hidalgo State. The number on each pie chart indicates the most abundant species, keys to Odonata species are in Table 2 (based and modified from HilleRisLambers et al. (2012)).

For suborder Zygoptera, the factor analysis (FA) suggests that only four factors (EV > 1) are needed to explain 83% of the model variance. The attributes that explained the variation were: TMN, HMN, HMX, pH, TDS, SAL and COND, so the remaining environmental variables can be eliminated in the canonical correspondence analysis. The trace obtained by the canonical correspondence analysis (CCA) is significant, indicating significant relationships between the environmental attributes and the presence of Zygoptera species (trace 1.798, p = 0.0257, inertia of the first two axes 13.51%, percentage of variance explained by the model 48.01%) (Fig. 5).

For suborder Anisoptera, the FA suggests that five factors (EV > 1) are needed to explain 93.4% of the model variance. The attributes needed to explain the variation were: TMN, TMX, HMN, HMX, pH, TDS, SAL and COND, so TA can be eliminated from the canonical correspondence analysis. The trace obtained by the CCA was not significant, so there are no significant relationships between the environmental attributes and the presence of Anisoptera species (trace 2.823, p = 0.8419, inertia of the first two axes 14.35%, percentage of variance explained by the model 46.04%) (Fig. 5).

For the total data, FA suggests that five factors (EV > 1) are required to explain 94.8% of the model's variance. The attributes needed to explain the variation were TMN, TMX, HMN, HMX, pH, TDS, SAL and COND, so TA can be eliminated in the canonical correspondence analysis. The trace obtained by the CCA is not significant, so there are no significant relationships between environmental attributes and the total presence of species (trace 2.343, p = 0.1514, inertia of the first two axes 12.65%, percentage of variance explained by the model 43.34%) (Fig. 5).

## Discussion

### Alpha and beta diversity

Contrary to our hypothesis, the CHD presented greater species richness than the other provinces. It was expected that species richness would be higher in the SMO and VE, as odonates are commonly known to prefer areas with high humidity and temperature, unlike the conditions in the CHD. This pattern of high species richness has also been observed for other animal groups, such as snakes, lizards and coleopterans (Fernández-Badillo et al. 2016, Canales and Goyenechea 2022, Cerón-Gómez et al. 2024). These findings support the importance of the CHD as an important area at the regional level for conservation of Odonata. The low species richness of odonates in the TMVB is attributed to adverse conditions limiting odonate diversity, except for certain species that can tolerate temperate temperatures and low humidity (Gómez-Anaya et al. 2011).

No significant differences in *^1^D* diversity were found amongst the biogeographic provinces, although some areas exhibited higher species richness. In some places, Anisoptera predominates, while Zygoptera shows different peaks in richness, influenced by specific environmental conditions of each province. However, relative abundances do not reflect variation in total abundances across the respective province (Kalkman et al. 2007).

Contrary to expectations, the two habitat types did not differ significantly in species richness and showed moderate dissimilarity. The samples were taken from year-round waterbodies, such as rivers and springs, which maintain high flows during the rainy season. During the dry season, flows are lower, but water reserves are maintained. For the suborders, a similar pattern was observed in Anisoptera, with higher species richness in lentic habitats (18 species) compared to lotic habitats (11 species). In contrast, Zygoptera exhibited comparable species richness between lentic and lotic habitats. These results are consistent with research from other parts of the world. Ait Taleb et al. (2024) reported 40% higher species richness in lentic habitats than in lotic habitats in a study conducted in Algeria. In temperate regions, such as the Nearctic and the Palaearctic, the number of lotic species is usually lower than that of lentic species (Dijkstra and Schröter 2020).

In Ghana, Seidu et al. (2019) observed that families, such as Calopterygidae and Aeshnidae, were found in lotic systems, with a strong affinity for canopy cover and fast-flowing waterbodies, whereas Libellulidae and Coenagrionidae were found in both lentic and lotic environments, but showed a strong affinity for lentic systems. Furthermore, lentic environments exhibit greater heterogeneity in species assemblages and are less predictable over time; therefore, lentic systems place greater pressure on species to adapt faster and disperse (Seidu et al. 2019).

Each biogeographic province contains species that were collected only in that province. CHD and VE had the largest number of unique species (11), while the pair of provinces with the highest number of shared species was SMO–CHD with five species in common. Only one species was present in all four provinces (*A. immunda*).

Throughout the studied provinces, the dominant species belong to the suborder Zygoptera and families Coenagrionidae and Calopterygidae. For the suborder Anisoptera, the dominant species belong to the family Libellulidae. At the global and national levels, the families Coenagrionidae and Libellulidae are the most widely represented. In the present study, the dominant species varied across provinces, potentially reflecting replacement and abundance influenced by the distinct environmental factors characterising each biogeographic province. In the TMVB, *I. demorsa* (Hagen, 1861) was the most abundant species (13 individuals) showing a clear preference for lentic areas. In the SMO, *A. ulmeca* (11 individuals) was found in both habitat types, while in the VE, *A. sedula* (16 individuals) was predominantly found in lentic habitats. This highlights the predominance of species associated with lentic areas, except for *H. calverti* in the CHD, which prefers lotic-type areas and was also the most abundant species overall (32 individuals) (Abbott 2011).

The biogeographic provinces also share a variety of species. *A. immunda* was found in all four provinces. This species has a wide distribution, extending from the USA to Belize and is recorded throughout much of Mexico. It is a generalist species with no habitat preferences, found in both lotic and lentic areas (Abbott 2011, Paulson et al. 2024).

The high dissimilarity between pairs of biogeographic provinces may reflect that biogeographic factors influence the high diversity of odonate species in the State.

Consistent with our hypothesis, a high degree of dissimilarity was observed amongst biogeographic provinces. These differences may be attributed to species that are exclusive to their respective provinces. Historically, the VE maintains a large number of species due to humid conditions, high temperatures and elevations of less 500 m above sea level, while species adapted to temperate climates and elevations above 1,000 m above sea level are commonly found in the TMVB (Alonso-EguíaLis et al. 2002, Silva-Hurtado et al. 2024).

The suborder Anisoptera was influenced by biogeographic province and the suborder Zygoptera was affected at the local level; this pattern has been documented in multiple studies worldwide. Anisoptera have higher tolerance to disturbance and greater dispersion capacity, enabling them to reach favourable habitats where they can reproduce. Zygopterans, on the other hand, are more sensitive to habitat perturbances or significant changes in some environmental variables. This sensitivity can lead to high dissimilarity amongst biogeographic provinces, driven by varying levels of disturbance, vegetation heterogeneity and environmental or water variables (De Marco Júnior et al. 2015).

Affinities for specific biogeographic components are reflected in species compositions. In the VE, species with Neotropical origin are observed, such as *H. titia*, while in the CHD, species of Nearctic origin are recorded, such as *P. longipennis*. In the SMO and the TMVB, species with a greater affinity for the MTZ, such as *B. pertinax* (Hagen, 1861) and *H. heterodoxum* (Selys, 1868), are recorded (Garrison 1990, Alonso-EguíaLis et al. 2002, Abbott et al. 2022).

Sampling localities belonging to the SMO are closer together, following the province's pattern of heterogeneity, exhibiting different vegetation and elevations that contribute to high species diversity. In contrast, the localities of the TMVB are more similar in elevation, climate and vegetation. Locality B1 is similar to the TMVB localities in terms of elevation and in the presence of forest. Therefore, it would be expected that the SMO and TMVB would share species of Anisoptera and Zygoptera from the family Coenagrionidae and be distinguished from each other by other Zygoptera species.

Localities A3 and B3 have a highly similar species composition, but different from that of other provinces due to low elevation, evergreen forests and high humidity, in contrast to localities A4 and B4 belonging to the CHD, where humidity is low and the presence of bodies of water limits the dispersal of certain species. For this reason, they share species of Anisoptera with dispersion ability and tolerance to high temperatures and low humidity. The dissimiliraty between these localities can be explained by the zygopterans with low dispersion ability, which are limited to staying in one place.

### Environmental relationships

The unique characteristics of each of the biogeographical provinces in the State of Hidalgo reflect a great diversity of odonates that are affected by the different factors in each of these areas at a regional level.

The exception was the SMO, which contains broad environmental heterogeneity. According to Morrone et al. (2017), the SMO belongs to the MTZ and contains multiple vegetation types, environmental heterogeneity and species diversity. This complexity lends great environmental variation to the SMO, increasing its similarities with other provinces in both abiotic and biotic factors.

As we hypothesised, most odonate species prefer areas with higher temperatures and humidity (Paulson and Haber 2021). These two factors are important, as they are positively related to diversity and richness. The highest species richness is found in tropical areas or, in exceptional cases, in montane cloud forests, which are characterised by relatively high humidity.

Water temperature influences the solubility of gases and contaminants, toxicity of chemicals, pH, density and electrical conductivity. Generally, higher temperatures promote microbial proliferation, metabolic activity and photosynthesis and affect the development and performance of biotic communities (Bonacina et al. 2022). Each species requires a particular temperature range for optimal performance. Species such as *A. percellulata*, *A. rhoadsi*, *E. umbrata*, *H. titia* and *O. discolor* showed positive correlations with higher water temperatures. In contrast, *I. denticollis*, *A. lacrimans*, *H. heterodoxum* and *S. illotum* were negatively correlated with temperature. Extreme temperatures can have adverse effects: high temperatures accelerate metabolism and speed up development, while low temperatures slow development and induce larval diapause until conditions become suitable for emergence (Norling 2021, Bonacina et al. 2022).

Conductivity (µS/cm) is associated with pH: areas with more acidic values tend to have higher conductivity, which promotes the diversity of generalist and monodominant species. Waterbodies with neutral pH exhibit lower conductivity, promoting species heterogeneity and ecological balance (Rychła et al. 2011). The pH recorded in most environments studied where multiple species were grouped was neutral to slightly alkaline (7 to 8), except for the lentic habitats of the SMO, where a pH of 5.5 was observed. Species such as *A. funebris*, *H. vulnerata* and *E. praevarum* were negatively correlated with conductivity.

High levels of total dissolved solids (TDS, ppm) are generally negatively correlated with odonate diversity and richness (Seidu et al. 2017, Sugiman et al. 2020). However, in the present study, higher richness and diversity were found in the CHD, where TDS values exceeded > 1000 ppm. In desert and semi-desert areas, waterbodies often exhibit high TDS values due to the accumulation of minerals, which, in turn, promotes the proliferation of organisms, such as bacteria, algae and other invertebrates, important food sources for odonates. In September, when water levels were higher, TDS values decreased, as they became more diluted. Some odonate species have been reported to have relatively high salinity tolerance. In general, odonates exhibit broad tolerance to salinity levels, meaning this factor does not greatly affect species richness and diversity (Berezina 2003). Most species were positively correlated with TDS and salinity, grouping near the centroid.

Zygopterans can be used for analysis at smaller scales. In this investigation, they showed more pronounced significant differences between biogeographic provinces due to sensitivity to the environmental variables at the local level. Anisoptera species respond to different variables at larger scales, given their high tolerance to the various type of disturbances that may occur in their habitats.

Most of the waterbodies where sampling took place were located in the Panuco hydrographic region. This region serves as an aquaculture zone, supplying water to the cities and towns in the State of Hidalgo. A smaller portion of the sampled area belongs to the Tuxpan-Nautla Region, which consists of multiple waterbodies that provide water to the tropical part of the State. It is important to study and protect these waterbodies and their biodiversity, as they are not part of or near any protected natural areas.

The evidence presented in this study shows that differences in dispersal ability do affect the effective size of the species pool of the two Odonata suborders, which is consistent with our hypothesis. The widespread Anisoptera species would follow a classical hierarchical community assembly mechanism, with environmental factors exerting their effects at large spatial scales and exhibiting a great capacity for dispersion within and between biogeographic provinces. In contrast, the effects of these environmental factors create a filter that may influence the local diversity of Zygoptera species, which exhibit smaller species ranges and lower limited capacity for dispersion, which is limited to certain areas. According to our data, biotic and abiotic factors operate interactively on both suborders, independent of scale. However, further research is needed to understand the role of biotic interactions in the composition of Odonata suborders species at regional and local scales.

The high dissimilarity amongst the studied biogeographic provinces highlights the need for conservation efforts, as these provinces contain few protected natural areas. A strategic approach is required to preserve the diversity of organisms associated with waterbodies, which are highly vulnerable to anthropogenic activities. The evolutionary differences between the biogeographic provinces foster significant diversity, placing the State of Hidalgo amongst Mexico’s top regions of odonate diversity. Continued research is essential in regions, such as the CHD and TMVB, where odonates thrive under challenging conditions. Odonates are positively related to parameters, such as humidity, environmental temperature and water temperature, which are crucial to their survival. In the case of Zygoptera species that show a strong dependence on local factors, it is crucial to conserve specific areas, given their limited dispersal capacity and sensitivity to changes in several environmental variables. Understanding the relationships between odonates and these environmental and water parameters is vital, as odonates are recognised as good indicators of both environment and water quality.

Odonate composition in the biogeographic provinces of Hidalgo is an interplay of biogeographical factors, environmental filters and dispersal abilities of each odonatan suborder. From a neutral perspective, this suggests that geographical barriers between provinces function as physical filters that restrict biotic flow. Consequently, the presence of a species in a specific province is because they successfully derived a function of their mobility capacity and persist adapting to the environmental conditions. Suborder Anisoptera has high mobility and better opportunities to colonise new waterbodies, making their distribution less predictable by local physicochemical variables and more dependent on regional connectivity and colonisation events (Brasil et al. 2017). Zygoptera have more restricted mobility, resulting in more stable species compositions on a local scale and intensifying the effects of environmental variables. Thus, Zygoptera species confined to specific lotic or lentic habitats are more vulnerable to stochastic disturbances, which could explain the high community dissimilarity between provinces (Brasil et al. 2017).

In localities with nearly identical physicochemical parameters, differences in species composition provide empirical support for the Neutral Theory, in which the environment is constant, but the species differ due to the dispersal capacities of each odonatan suborder. The prevailing mechanism is dictated by historical biogeographical conditions for diversity patterns, but environmental factors explain dissimilarity amongst waterbodies. However, the environmental filtering hypothesis gains more strength, since the most distant locations are at a distance of ~ 150 km, which, in biogeographical scales and terms, is a relatively short distance. Further studies should include this approach to better understand the factors modelling the distribution of odonate species in waterbodies at different scales.

## Conclusions

The structure of the assemblages is driven by the interaction between biogeographic history and local environmental variables, in which species composition across provinces is influenced by factors, such as temperature and humidity, which are determinants of diversity.

A differentiated response was identified across taxonomic groups; while species of suborder Anisoptera exhibit greater tolerance and dispersal capacity at broad scales, Zygoptera act as habitat specialists due to lower dispersal capacity and are sensitive to local changes in the ecosystem and water. The high beta diversity observed amongst biogeographic provinces indicates that each region maintains a unique assemblage, characterised by high species replacement rather than nestedness. The Chihuahuan Desert (CHD) and the Sierra Madre Oriental (SMO) act as distinct evolutionary areas in which environmental filtering affects community composition, resulting in low biotic similarity despite their geographic proximity. This underscores the importance of the Mexican Transition Zone as a complex mosaic where Neotropical and Nearctic elements converge and the ecological importance of arid zones, such as the Chihuahuan Desert, for odonates is underestimated.

While environmental variables, such as temperature and humidity, establish specific tolerance thresholds for certain species, neutral dynamics may ultimately determine which members of the regional species pool effectively occupy each province. This is particularly evident in a region as complex as the Mexican Transition Zone, where a convoluted geological history has fragmented populations and amplified the role of stochastic processes. Understanding the relationships amongst odonates, environmental factors, water parameters and neutral components is vital for designing strategies to preserve the diversity of these organisms associated with waterbodies that are highly vulnerable to anthropogenic activities. It is urgently neccesary to conserve the integrity of habitats (lentic and lotic), ensuring the persistence of odonates as bioindicators of ecosystem health in the Mexican Transition Zone.

## Figures and Tables

**Table 1.** Biogeographic province, coordinates and characteristics of sampling locations.

**Biogeographic province**	**Code**	**Locality**	**Latitude**	**Longitude**	**Elevation (m a.s.l)**	**Climate**	**Vegetation types**	**Hydrology**
Sierra Madre Oriental (SMO)	A1	Xochiacoatlán, Tianguistengo	20.7525	-98.6512	1020	Temperate	Tropical forest and semi-evergreen medium forest	Year-round rivers and springs
	B1	Poza Honda, Agua Blanca Iturbide	20.3659	-98.4141	2230	Cold temperate	Coniferous forest, montane cloud forest, and farmland vegetation	Several year-round rivers (Pánuco, Cazones and Tecolutla)
Trans-mexican Volcanic Belt (TMVB)	A2	Tezoncualpan, Cuautepec de Hinojosa	19.9329	-98.2594	2640	Cold temperate	Pine, oak, and oyamel fir forest with various herbaceous plants and shrubs	Tributaries that flow in the Tecocomulco Lagoon
	B2	Coatlaco waterfalls, Apan	19.7869	-98.367	2620	Cold	Pine, oak, oyamel fir and xerophilous scrub	Belongs to the Moctezuma River Basin and has various rivers, streams and lagoons
Veracruzan (VE)	A3	Coapantla River, Huautla	20.9844	-98.2461	150	Extremely hot climate	Low deciduous forest	Mainly streams and rivers (San Gregorio, Pantepec etc.).
	B3	Pantepec River, San Antonio el Grande, Huehuetla	20.4727	-98.0341	365	Warm climate	Tall evergreen forest vegetation and farmland	Four main rivers flow through the area (Chiflón, Huehuetla or Pantepec, Blanco and Beltrán).
Chihuahuan Desert (CHD)	A4	Manantial El Huemac, Tasquillo	20.5193	-99.3744	1735	Temperate climate	Scrubland and mesquite vegetation predominating	Major rainfed rivers are the Pánuco River, Moctezuma River, Tula River and springs such as El Huemac.
	B4	Moctezuma River, Uxdejhé, Tecozautla	20.5832	-99.6822	1640	Temperate climate	Xerophilous scrub vegetation and small oak and oyamel fir forests	Dam, wells, springs and rivers including Pánuco, Tecozautla, San Francisco and Moctezuma.

**Table 2.** Taxonomic list of species collected in the biogeographic provinces studied. Classification according to Paulson et al. (2025) (*new state record; Abbreviation indicates the habitat type where it was captured: LE = lentic, LO = lotic, G = both habitats).

**Key**		**CHD**	**TMVB**	**SMO**	**VE**
	**Suborder Zygoptera**				
	**Superfamily Lestoidea, Family Lestidae**				
1	*Archilestes grandis* (Rambur, 1842)		LE	LE	
	**Superfamily Calopterygoidea, Family Calopterygidae**				
2	*Hetaerina americana* (Fabricius, 1798)	LE			
3	*Hetaerina calverti* Vega-Sánchez, Mendoza-Cuenca & González-Rodríguez, 2020	LO			
4	*Hetaerina cruentata* (Rambur, 1842)			G	
5	*Hetaerina infecta* Calvert, 1901			LO	
6	*Hetaerina occisa* Hagen *in* Selys, 1853				LO
7	*Hetaerina titia* (Drury, 1773)				LO
8	*Hetaerina vulnerata* Hagen *in* Selys, 1853	LO		LO	
	**Superfamily Coenagrionoidea, Family Coenagrionidae**				
9	*Argia anceps* Garrison, 1996	G		G	LE
10	*Argia barretti* Calvert, 1902				LO
11	*Argia funebris* (Hagen, 1861)	G		G	
12	*Argia immunda* (Hagen, 1861)	G	LO	LE	LO
13	*Argia munda* Calvert, 1902	G	LE	LO	
14	*Argia nahuana* Calvert, 1902	LE			
15	*Argia lacrimans* (Hagen, 1861)			G	
16	*Argia oculata* Hagen *in* Selys, 1865	LE			
17	*Argia oenea* Hagen *in* Selys, 1865	LO			G
18	*Argia percellulata* Calvert, 1902				LE
19	*Argia rhoadsi* Calvert, 1902				LO
20	*Argia sedula* (Hagen, 1861)				G
21	*Argia translata* Hagen *in* Selys, 1865	G		LE	G
22	*Argia ulmeca* Calvert, 1902			G	
23	*Enallagma civile* (Hagen, 1861)	LE			
24	*Enallagma novahispaniae* Calvert, 1907	G		LE	LE
25	*Enallagma praevarum* (Hagen, 1861)	G	G	LE	
26	*Hesperagrion heterodoxum* (Selys, 1868)		LE	LE	
27	*Ischnura demorsa* (Hagen, 1861)	LE	G		
28	*Ischnura denticollis* (Burmeister, 1839)		G		
29	*Telebasis salva* (Hagen, 1861)	G			LE
	**Suborder Anisoptera**				
	**Superfamily Aeshnoidea, Family Aeshnidae**				
30	*Anax junius* (Drury, 1773)	G			
31	*Rhionaeschna multicolor* (Hagen, 1861)		LO		
32	*Rhionaeschna jalapensis* (Williamson, 1908)	G			
	**Superfamily Gomphoidea, Family Gomphidae**				
33	*Erpetogomphus crotalinus* (Hagen *in* Selys, 1854)	LO			
34	*Phyllogomphoides albrighti* (Needham, 1950)				LO
35	*Phyllogomphoides suasus* (Selys, 1859)			LO	
	**Superfamily Libelluloidea, Family Libellulidae**				
36	*Brechmorhoga vivax* Calvert, 1906				LO
37	*Brechmorhoga pertinax* (Hagen, 1861)			LO	
38	*Cannaphila vibex* (Hagen, 1861)	LE			
39	*Dythemis nigra* Martin, 1897			LE	
40	*Dythemis nigrescens* Calvert, 1899	LE			G
41	*Erythrodiplax umbrata* (Linnaeus, 1758)				LE
42	*Libellula croceipennis* Selys, 1868	LE		LE	LE
43	*Libellula saturata* Uhler, 1857	G	LE		
44	*Macrothemis inacuta* Calvert, 1898				LE
45	*Macrothemis pseudimitans* Calvert, 1898			LO	G
46	*Orthemis discolor* (Burmeister, 1839)				LE
47	*Pachydiplax longipennis* (Burmeister, 1839) *	LE			
48	*Pantala flavescens* (Fabricius, 1798)	LE			LE
49	*Perithemis intensa* Kirby, 1889	LE			
50	*Sympetrum corruptum* (Hagen, 1861)	LE			
51	*Sympetrum illotum* (Hagen, 1861)		LE	LE	

**Table 3.** Average values of environmental variables from biogeographic provinces sampled in the State of Hidalgo, México (TMN = Minimal ambient temperature, TMX = Maximum ambient temperature, HMN = Minimum humidity, HMX = Maximum humidity, TA = Water temperature, TDS = Total dissolved solids, SAL = Salinity, COND = Conductivity).

Habitat	Variable	Biogeographic province			
		SMO	TMVB	VE	CHD
AIR	TMN (°C)	22	17	20	28
	TMX (°C)	33	28	25	35
	HMN (%)	20	10	41	12
	HMX (%)	67	31	70	30
LENTIC	pH	7	8	7	7
	TA (°C)	19	11	23	22
	TDS (ppm)	97	78	139	415
	SAL (%)	0.1	0.1	0.2	0.05
	COND (µS/cm)	197	71	205	802
LOTIC	pH	7	8	7	8
	TA (°C)	18	14	22	21
	TDS (ppm)	79	65	105	395
	SAL (%)	0.08	0.06	0.1	0.04
	COND (µS/cm)	160	130	212	793

**Table 4.** Pair-wise comparison analysis between biogeographic provinces in Hidalgo State using Bonferroni test , T-F statistic; p (perm) - p value obtained with permutations.

**Total**		
	F	p
**CHD-TMVB**	9.262	0.0012
**CHD-SMO**	4.384	0.003
**CHD-VE**	6.624	0.0018
**TMVB-SMO**	4.275	0.0012
**TMVB-VE**	6.431	0.0006
**SMO-VE**	2.811	0.0024
** Anisoptera **		
	F	p
**CHD-TMVB**	9.271	0.0006
**CHD-SMO**	5.432	0.0024
**CHD-VE**	11.31	0.0018
**TMVB-SMO**	3.387	0.003
**TMVB-VE**	5.545	0.0006
**SMO-VE**	2.7	0.0108
** Zygoptera **		
	F	p
**CHD-TMVB**	12.02	0.0018
**CHD-SMO**	5.005	0.0018
**CHD-VE**	8.577	0.0024
**TMVB-SMO**	4.902	0.0024
**TMVB-VE**	7.824	0.0048
**SMO-VE**	3.253	0.0024
